# Cause and Management of Non-Specific Infection of the Extremities

**Published:** 1908-05

**Authors:** L. Sexton

**Affiliations:** Minor Surgery, Tulane Univ. Med. Dept.


For the Texas Medical Journal.

### Cause and Management of Non-Specific Infection of the Extremities.

BY L. SEXTON, M. D., MINOR SURGERY, TULANE UNIV. MED. DEPT.

The term infection applies to morbid condition caused by the entrance and growth within the body of pathogenic micro-organisms, and to the act or process by which disease is thus produced (Keen).

The term infection is applicable alike to wounds which contain bacteria and to conditions in which the germs enter into the circulation of the blood. The pyogenic infections result in sapremia, local, or in septicemia, when the germs enter the circulation. When arrested in internal organs, as the liver, spleen or in the lymphatic glands, producing abscesses, we have the condition known as pyemia or metastatic abscess, but it is not with this more serious phase of the trouble with which this paper has to deal, as it is intended to offer some suggestions in the treatment or management of ordinarily infected wounds of the extremities.

Bacteria enter the system through the mucous membrane and abrasions of the skin. The abrasion may not be larger than a pin point or a hair follicle. If the phagocytic action of the patient is reduced by disease, or decreased by the virulence of the germs, the consequence is more serious. When the infecting germs remain localized and the ptomains are only absorbed it produces a condition known as bacterial intoxication.

The infection following these abrasions to the skin are termed pyogenic or non-specific infections. They result in inflammatory reaction, which produce the molecular death of the part, ulceration, gangrene, or in liquifaction of tissue and inflammatory material resulting in pus or suppuration, or when properly treated at first by rest and large wet antiseptic gauze applications or hot drip, it may result in resolution.

The above described process is the pathological story of the ordinary acute abscess or infection.

After the pus has become circumscribed and evacuated there is very little left to be done, but when these organisms invade the cellular tissue producing toxemia, or when the bacteria invade the blood stream and become lodged in different internal organs or glands, giving rise to mestastatic abscess, pyemia or septicemia, it is quite a different proposition.

These are the conditions that the surgeon has mostly to fear, and it should always be remembered that the severest attack of these troubles had for its beginning a very trivial cause.

*Cause.*—The staphylococcus pyogenes is found practically everywhere that micro-organisms are capable of existing, but its presence in numbers in the atmosphere, under finger nails, upon the skin, within the cavities of the body and upon the many objects and tools coming in contact with persons, makes this organism of special interest to the surgeon.

It is estimated that 15 per cent of all germs found in the atmosphere of amphitheaters and hospital wards are the staphylococci pyogenes aurens. This variety of organisms are not so frequently found in the ordinary water about premises. It is known as the pus germ and is the cause of perhaps 90 per cent of abscesses and infections. It is nearly always found as one of the germs in all mixed infection. The staphylococcus pyogenes aurens penetrates the epithelium through the hair follicles, or through abrasions of the skin. The sweat glands are not often involved but the osseous system is frequently invaded.

Pustulæ, acne, furuncles and boils are produced by the staphylococcus. The same germ is at the bottom of most suppurative cases following operations.

Streptococci also have a very wide dissemination in the atmosphere and is the cause of hospital wards becoming infected with erysipelas and other severe infectious diseases.

Surgeons coming in contact with this germ may convey them from the hospital under their finger nails or on their instruments, and may thereby infect the next patient whose wound they dress, provided the most thorough asepsis has not been practiced.

Streptococci are found upon the skin of our bodies, in the nostrils, upon the tonsils, within the cervix uteri and many other portions of the anatomy. They have been found in the blood and urine when no general infection was present.

The prominent feature of the streptococci is the production of hemolysin, a non-poisonous substance, which has the property, however, of dissolving red blood corpuscles. The streptococci is highly pathogenic. It is the cause of erysipelas, furunculosis, abscess, lymphangitis, angina, phlegmon, tonsilitis, enteritis, septicemia, pyemia, osteomyelitis, rheumatism and myelitis. (Keen.)

It is often associated with mixed infections as dyphtheria, scarlet fever, tuberculosis and other infectious diseases.

The bacilli erysipelatus and streptococci are the same germ; they produce infection or erysipelas according to a not well understood factor of differing resistance of the individual patient, size, location and depth of the wound into the body and the varying virulence of the cultures.

Streptococci has an affinity for the lymph vessels and spaces through which it travels very rapidly, provided there is any squeezing or massage of the part. Streptococci is not obstructed in its progress by fixed tissue cells, barriers or leukocytes, as is the staphylococci. Its leukotactic powers being feeble. The production of liquifaction of tissue and production of pus is also small, though its presence is accompanied by the most violent inflammatory processes.

The pus produced by streptococci is usually scant and watery; if an abundance of yellow, creamy pus is found you may be sure of a mixed infection with staphylococci, as is well illustrated in phlegmonous erysipelas. Inflammations in which streptococci exists have a tendency to spread widely, the streptococci preparing the way, as it were, for the staphylococci, after which the streptococci may practically disappear, as is the case in many abscess cavities. Purely staphylococci suppurations are generally more limited, being circumscribed by a hemmed in wall of leukocytes.

*Symptoms.*—Although the general symptoms in streptococci infections are more violent and marked, there is nothing absolutely to diagnose them from those arising from the staphylococcus and from other bacterial infections, except in degree.

The marked up and down temperature is not pathognomonic, as has been stated by some authors. The temperature, however, is usually higher and more irregular with rapid pulse, hard breathing, coated, dry tongue and delirium in the severer cases of streptococcic infections.

*Treatment.*—All the prophylactic measures such as cleansing of the wounds, hands and instruments that are recommended in aseptic surgery should be carried out in its minutest detail.

Sterilized water and salt and boracic acid solutions usually serve as good purpose for irrigation, as do the stronger germicidal agents, which in many instances, if used strong, destroy the tissue, coagulating the albumen, as well as any destructive action they may possess on the germs present.

The skin surrounding the infected wound should be shaved, if it is covered with hair, should be wiped off with alcohol or spirits of turpentine, and thoroughly scrubbed, if not too tender, with soap and water. This should be followed by rinsing off with 1-2000 bichloride solution. If the wound is superficial peroxide of hydrogen poured over, as it oxidizes pus and necrotic tissue, after the peroxide, flush with sterilized saline solution.

If it is a hand or foot that is covered with machinery grease, dirt or grime, it is utterly impossible to make it perfectly clean at the first dressing without an anesthetic.

The horny hand of the laboring man is best softened by what might be called the surgical aseptic poultice or an abundance of sterilized gauze kept wet with either one-half of 1 per cent of carbolic acid solution, or 1 to 5000 of bichloride solution.

Don't send the patient away until you have furnished him with these antiseptic solutions with which to keep the wound moist for the first twenty-four or forty-eight hours, as by this time you will find much of the horny layer so softened that the cleansing and the rendering of the hand aseptic is much more easy to accomplish.

We can either keep up this moist dressing, or after this we can adopt either the ordinary antiseptic dusting powders, or some of the antiseptic ointments with 10 per cent ichthyol, provided there is much burning inflammation and pain connected with the wound. Tincture of iodine is an excellent germicidal application in such conditions. Local applications of thirty grains chloride of zinc to the ounce of water, or pure carbolic acid immediately followed by the alcohol may be required for some cases in which the infection has been of a most malignant type. All retention sutures, manipulation of the part, and cutting away of any except absolute necrotic tissue should not be recommended, from the fact that it opens up that many more sources of infection.

After cellulitis has begun, the use of large, hot, moist applications will assist in producing resolution or in relieving the severity of the disease by keeping the capillary circulation open.

If the phlegmonous process of inflammation or cellulitis continues, threatening gangrene or sloughing of the part, numerous and free longitudinal incisions are to be made. Under such conditions it is best to use local anesthesia if the infection has weakened the patient too far for it to be safe to give a general anesthetic which may lessen his resistance to sepsis. Moist dressings in the form of hot applications are preferable to dry powders, because the moist dressing prohibits the drying of the discharges and interference with absorption. When extremities have been incised in a more virulent form of infection, if they can be placed in a continuous hot bath as the ordinary foot tub for either arm or leg without any dressing whatever, the capillary circulation is much improved as well as the relief obtained by the hot application, continuous drainage is better obtained in the bath than by bandaging.

*Prevention.*—Remembering that we have such an infinitesimal micro-organism common to all the dirt and grime with which the hands and tools of the average laborer is infected, it becomes our province upon the least abrasion of any parts to so thoroughly cleanse the wound with antiseptic solutions, that any germs being left alive is practically impossible. When the field is once made aseptic, then keep it so by less frequent dressings and more constant applications of the standard antiseptic solutions.

Very often these wounds are inflamed and septic before they come under the care of the physician. The lymphatic vessels and veins are involved and some cases have even gone on to purulent discharges and destruction of tissue.

In the management of these cases, even though already infected, we should use the same aseptic precautions about our hands and instruments as we would in a fresh surgical operation, lest we add another burden of responsibility by making a mixed infection of a case that is already bad enough. To prevent carrying the infection to another patient it is better to dress such cases with hands protected by rubber gloves.

The majority of germs, even in an abscess cavity, are dead, but the products of their decomposition carries on the inflammation just as though they were living entities. This gives a cue to the treatment of all such infections. Thorough drainage should be the slogan in every infected wound. I have been led to believe that excessive irrigation and injections of peroxide of hydrogen into sinuses and infected cavities is not a good plan of procedure, as by this very method we may drive these germs on into non-infected areas.

As a matter of course the ordinary cleansing of the part with some germicidal irrigation would seem the proper thing to do, yet all of us understand that there are always germs left after the most thorough bichloride irrigation.

The hot carbolic drip for twenty-four or forty-eight hours is one of the best methods of subduing these infections, because it penetrates into every portion of the diseased tissues. I have recently saved a foot (compound fracture) by this hot drip.

Any antiseptic solution strong enough to destroy all the germs would also destroy the tissue which contain the germ. They are neither desirable or necessary and should be used only in moderate strength and in first aids. Rest of the extremity is absolutely important; it should be obtained in the leg by putting the patient to bed with the extremity elevated, and in the hand and arm by applying a splint and swing and allowing no manipulation of the part, which only lights up and spreads the germs to non-infected areas.

The Biers method of applying a light bandage around infected extremities just tight enough to cause a slight blueness for an hour or so through the day has been an invaluable benefit in many of my cases.

The general treatment consists in the meeting of symptoms as they arise, such as sponging and ice applications for excessive temperature, strychnia and digitalis for weak, fast hearts, the keeping of the kidneys and bowels flushed out well by any suitable remedies and an abundance of water. The Murphy drip per anum, in order to have the patient take up more fluids, thus diluting the ptomains, are all valuable adjuncts in bringing these severe infections to a successful termination.

With acne infections, Wright's bacterial staphylococci vaccination has proven a great success in some cases, and its further field of usefulness is one of the future helps to be looked forward to in the treatment of all these bacterial infection.

Most cases of acne boils and common pus infection of wounds is due to staphylococcus. Any of these when chronic may be treated with bacterial vaccine, according to Prof. Wright's opsonic theory. Though the opsonic index is desirable, and no doubt often a vaccine made from the germ in the particular case would give better results, it is perfectly practical to give a patient a mixed staphylococcus vaccine, which can now be obtained from the pharmaceutical houses or from any good bacteriological laboratory.

The ordinary dose of staphylococcus vaccine is 200 to 1000 million of dead germs, and the interval between doses one week to ten days. The technic of giving the vaccine does not differ from that of giving any other hypodermic injection.

